# Supplementary material for: Enhancing Minds in Motion® as a virtual program delivery model for people living with dementia and their care partners
Source: PLoS One. 2024 Jan 19;19(1):e0291166. doi: 10.1371/journal.pone.0291166 (PMC10798436; doi:10.1371/journal.pone.0291166)
Supplement: S1 File — (DOCX) [file pone.0291166.s001.docx]

**Evaluation of the Minds in Motion Virtual Program**

**Post-Program Semi-Structured One-on-One Interview: Guiding Questions (Participants)**

1. Tell me about your experience in the virtual Minds in Motion program.
   - What was the program like for you?
   - What did you like best about the program?
   - What, if anything, did you not like about the program?
   - How did participating in the program make you feel?
   - Did you experience challenges participating in the program? If so, what challenges did you experience?
   - What helped support you to participate in the program (your care partner, exercise instructor, volunteers)?
   - How was your experience in the virtual Minds in Motion program different than your experience in the in-person Minds in Motion program?
2. How has participating in the program had an impact on you (your life, abilities, or perspectives)?
   - How has the program benefited you?
   - How do you think the program can benefit people with dementia in the community?
   - How has this program impacted your care partner?
   - How has participating in the program changed your perspective on dementia?
3. How has participating in the program helped you to live well?
   - How has the program helped you to be physically active?
   - How has the program helped you to feel socially connected? What relationships or friendships have you made in the program?
4. What recommendations do you have for making the program activities better?
   - What could be done to improve the exercise portion of the program?
   - What could be done to improve the mental and social stimulation activities?
   - What other activities would you like to see?

5. What recommendations do you have for making the program processes better?

- - What could be done to make the orientation session better or more helpful?
  - How could the virtual delivery of the program be improved?
  - Are you satisfied with two sessions per week? How did you like the current length of the program (8 weeks)?

6. What else you would like to share about your experience in the virtual Minds in Motion program?

7. If this program were to be offered again in your community would you join:

- In person (yes/no)
- Virtual (yes/no)
- Which would you prefer, if either, and why?

8. Do you have any questions for me?
