# Supplementary material for: Enhancing Minds in Motion® as a virtual program delivery model for people living with dementia and their care partners
Source: PLoS One. 2024 Jan 19;19(1):e0291166. doi: 10.1371/journal.pone.0291166 (PMC10798436; doi:10.1371/journal.pone.0291166)
Supplement: S2 File — (DOCX) [file pone.0291166.s002.docx]

**Evaluation of the Minds in Motion Virtual Program**

**Program Evaluation**

- 1. The comments provided within this survey are anonymous (your name will not be shared) and will not impact your relationships or participation in the program.

1. Please rate your level of level of agreement with the following statements, with 1 being that you strongly disagree and 5 being that you strongly agree.

|  | 1  Strongly Disagree | 2  Disagree | 3  No Opinion | 4  Agree | 5  Strongly Agree |
| --- | --- | --- | --- | --- | --- |
| a. I enjoyed participating in the Minds in Motion virtual program. |  |  |  |  |  |
| b. I feel that I have benefited from the virtual Minds in Motion program. |  |  |  |  |  |
| c. I feel I was treated with respect while participating in Minds in Motion. |  |  |  |  |  |
| d. I feel that the program leaders did a good job leading the program. |  |  |  |  |  |
| e. I felt comfortable asking questions or sharing concerns with the staff. |  |  |  |  |  |
| d. I enjoyed the physical activity part of Minds in Motion. |  |  |  |  |  |
| e. I enjoyed the cognitive and social stimulation part of Minds in Motion. |  |  |  |  |  |
| f. I felt comfortable taking the MiM program in a virtual setting. |  |  |  |  |  |
| g. I was satisfied with the length of the class. |  |  |  |  |  |
| h. I was satisfied with the number of sessions per week. |  |  |  |  |  |
| i. I would recommend the virtual Minds in Motion program to other people. |  |  |  |  |  |

2. How would you rate your overall experience in the virtual Minds in Motion program

|  | Poor | Fair | Good | Very Good | Excellent |
| --- | --- | --- | --- | --- | --- |
| Overall Experience |  |  |  |  |  |

3. How would you rate the difficulty of the program?

|  | Too Hard | Somewhat Hard | Just Right | Somewhat Easy | Too Easy |
| --- | --- | --- | --- | --- | --- |
| Program Difficulty |  |  |  |  |  |

4. If I could change anything about the Minds in Motion program it would be…
