# Supplementary material for: Enhancing Minds in Motion® as a virtual program delivery model for people living with dementia and their care partners
Source: PLoS One. 2024 Jan 19;19(1):e0291166. doi: 10.1371/journal.pone.0291166 (PMC10798436; doi:10.1371/journal.pone.0291166)
Supplement: S3 File — (DOCX) [file pone.0291166.s003.docx]

| **Minds in Motion Observation Checklist ___________/_____/____** *year/month/day*  **___________ - _____________** *program time* | |
| --- | --- |
| # of participants present | Persons with dementia: ______  Care partners: ______ |
| # of participants absent | Persons with dementia: ______  Care partners: ______ |

| **Instructor and Volunteer Training and Qualifications** | **No** | | **Yes** | **NA** |
| --- | --- | --- | --- | --- |
| Physical activity instructor(s) has valid exercise certification/designation or training |  | |  |  |
| Instructors have completed emergency procedures training within the last year |  | |  |  |
| Volunteers have completed emergency procedures training within the last year |  | |  |  |
| Volunteers have received volunteer program training within the last year |  | |  |  |
| **Notes:** | | | | |
| **Program Practices** | **No** | | **Yes** | **NA** |
| Instructors and volunteers connected prior to class start for team huddle |  | |  |  |
| Program attendance is completed |  | |  |  |
| ***Notes:*** | | | | |
| **Safety** | **No** | | **Yes** | **NA** |
| ≥2 staff/volunteers (at least one being a staff member) present for class |  | |  |  |
| ≤10 participants with dementia attending the class |  | |  |  |
| Safety procedures reviewed at the start of class |  | |  |  |
| Feedback and suggestions for the participants’ environment is given if needed |  | |  |  |
| All participants had their webcams turned on |  | |  |  |
| Care partners for each participant with dementia was participating or nearby OR exceptions were pre-approved |  | |  |  |
| At least two variations of each exercise demonstrated by staff/volunteers |  | |  |  |
| Instructors identified signs/symptoms that required participant(s) to stop exercising  Signs/Symptoms identified by instructor included: ___________________________________________________________________ |  | |  |  |
| ***Notes:*** | | | | |
| **Program Delivery** | **No** | **Yes** | | **NA** |
| Program started on time (+/- 5min) Start Time: _____________ |  |  | |  |
| Program ended on time (+/- 5min) End Time: _______________ |  |  | |  |
| Instructor followed the mental and social stimulation activities plan |  |  | |  |
| Transitions between class components were smooth |  |  | |  |
| **Notes:** | | | | |

| **Class Component** | **Exposure**  **Planned vs Actual** | | **Adherence**  **Yes/No/NA** | **Quality**  1=Low (confusing)  3=Medium  5=High (clear) | **Participant Responsiveness**  1=Low (unengaged)  3=Medium  5=High (engaged) |
| --- | --- | --- | --- | --- | --- |
| Participant welcome | **3-5 min** |  |  |  |  |
| Safety review | **1-2 min** |  |  |  |  |
| Exercise | **40-45 min** |  |  |  |  |
| Mental and social stimulation | **30-45 min** |  |  |  |  |

| **Physical Activity Instructor Top Behaviours**  **Name:** | ***Observed***  ***Yes/No/NA*** | ***Quality***  1=ineffective  3=Medium  5=Highly Effective |
| --- | --- | --- |
| Listens attentively when interacting with participants |  |  |
| Promotes a positive environment |  |  |
| Observes and corrects participants’ form, posture and breathing as needed |  |  |
| Demonstrates exercises correctly to participants |  |  |
| Interacts and initiates direct contact with all participants |  |  |
| Acts and advises only within scope of program |  |  |
| Tracked intensity of exercise via RPE |  |  |
| Achieved a moderate to vigorous physical activity level for most participants |  |  |
| Achieved a full body workout |  |  |
| Integrated aerobic, resistance, and balance training |  |  |

| **Mental & Social Stimulation Activities Instructor Top Behaviours**  **Name:** | ***Observed***  ***Yes/No/NA*** | ***Quality***  1=ineffective  3=Medium  5=Highly Effective |
| --- | --- | --- |
| Listens attentively when interacting with participants |  |  |
| Promotes a positive environment |  |  |
| Effectively guides participants through activities |  |  |
| Demonstrates activities correctly to participants |  |  |
| Interacts and initiates direct contact with all participants |  |  |
| Acts and advises only within scope of program |  |  |
| Activities align with recommendations for cognitive stimulation |  |  |

| **Volunteer Top Behaviours**  **Name:** | ***Observed***  ***Yes/No/NA*** | ***Quality***  1=ineffective  3=Medium  5=Highly Effective |
| --- | --- | --- |
| Listens attentively when interacting with participants |  |  |
| Promotes a positive environment |  |  |
| Cultivates group support and introduces new participants to other participants and members |  |  |
| Acts and advises only within scope of program |  |  |
| Provided technology support when/if required |  |  |
