# Supplementary material for: Enhancing Minds in Motion® as a virtual program delivery model for people living with dementia and their care partners
Source: PLoS One. 2024 Jan 19;19(1):e0291166. doi: 10.1371/journal.pone.0291166 (PMC10798436; doi:10.1371/journal.pone.0291166)
Supplement: Attachment — Submitted filename: Response to Reviewers.docx [file pone.0291166.s006.docx]

**Title:** Enhancing Minds in Motion^®^ as a virtual program delivery model for people living with dementia and their care partners

(Submitted to PLOS One)

**Response to Reviewers**

Thank you for the opportunity to respond to reviewer comments. We appreciate the reviewers’ insights and have edited the manuscript using their feedback. We have listed the reviewer comments below in bold text, followed by our response in regular text.

**Reviewer: Academic Editor**

**Comments to the Author**

**Why did you decide to do pre-post assessment before and after 2 weeks? Pre-assessment I agree, however for the post assessment, is there any justification of selecting two weeks interval?**

Thanks for this question. As we were aiming to evaluate the direct impact of the program (and not sustained benefits), we aimed to assess outcomes as soon as possible after the intervention. Given that all participants in a program session completed the program at the same time, we needed a 2-week interval to fit in all the assessments with our assessor. This was especially important for the larger classes. In many cases post-assessments were completed within 1 week of a participant completing the program, but there were situations where the participant couldn’t meet with a research assistant within the week due to holidays or scheduling difficulties.

**Also, I don't think you could conclude based on one post assessment soon after intervention. Any plans to assess the sustainability?**

We were not funded to assess sustainability in this study. In addition, a complication of assessing the sustainability of a single program offering is that a high percentage of participants re-enrolled immediately in a subsequent session of Minds in Motion. In our initial ramp up, we did do some assessments with people who were doing the program for a second time. These indicated smaller improvements in quality of life in the second offering, but certainly sustained benefits. When the project was fully ramped up, we no longer had capacity to fit in assessments of second-time participants so have insufficient data for publication.

**In programme or project evaluation, there should be criteria for evaluation and also agreed timeline.**

We agree that program evaluations should have specific criteria and an agreed upon timeline. Indeed, we aligned our program evaluation with the logic model for our funding program (the Public Health Agency of Canada Dementia Community Investment) and assessed these outcomes immediately post-intervention. While the logic model is not publicly available, the intermediate outcomes were improved health behaviours, which we evaluated with the International Physical Activity Questionnaire and Cognitive Leisure Activity Scale, and protective factors (social isolation/engagement), which we evaluated with the Social Provisions Scale. The ultimate outcomes were improvements in wellbeing, which we evaluated with the DEMQOL and several different scales for care partners. We also include details for the timeline for the project, number of program sessions offered, number of people participating in those programs, and number of people participating in the evaluation.

**You mention that you find out acceptability and impact on QOL. Did you think about the effectiveness of the intervention?**

Thanks for this question. Yes, we were interested in the effectiveness of the evaluation on health behaviours and quality of life/wellbeing. These were the primary outcomes aligned with the National Dementia Strategy, and its implementation within this funding program. We have demonstrated this in the last sentence of our introduction: The purpose of the manuscript is to describe virtual MiM and evaluate it's feasibility and effectiveness.

**Also, I think it's too early to do the post assessment in two weeks.**

We note your concern but would politely disagree. Most evaluations of physical activity and lifestyle interventions include an immediate post-program evaluation to assess the effectiveness of the program. We do agree that it would be valuable to also understand the sustained impact over time, should additional support for evaluations become available. The challenge in doing so in a publicly available program is that many participants re-enrol immediately in a subsequent Minds in Motion offering so it is difficult to capture a post-program window with no intervention. It is likely that they will experience greater benefits from repeat enrolments than enrolment in a single offering over a period of time.

**DEMQOL which you used for QOL measurements has several components, and also its a complex measure of QOL which gives overall well-being. QOL is a psychological construct which is multifactorial. Do you think about the other confounding effects when selecting the sample?**

Our intention in using the DEMQOL was to capture overall well-being. For care partner, we had also started with a global measure of quality of life/wellbeing, but objections to some of the questions from our advisory team members led us to use several scales instead.

We specifically set very broad inclusion criteria for our sample to accurately reflect the people who enrol in the program as it is run in the community. Our only restrictions were safety to exercise and sufficient cognitive capacity to participate in the discussions and evaluation. Of course, those who enrol in the study are not representative of the broader population. They are more likely to be white, female, with high education, for example, which we expected as these individuals are more likely to seek out and use community programs and services (Elavsky et al., 2017; Carter et al., 2020).

We did some post hoc exploration of demographic factors that predict the magnitude of changes in quality of life but had limited confidence to make conclusions due to low sample sizes. This is an important area for future evaluation.

Elavsky S, Smahel D, Machackova H. Who are mobile app users from healthy lifestyle websites? Analysis of patterns of app use and user characteristics. Transl Behav Med. 2017;*7*(4):891–901. https://doi.org/10.1007/s13142-017-0525-x

Carter A, Alexander AC. A qualitative exploration of women’s experiences who belong to a “fitness community.” Am J Health Educ. 2020;51(1):22-30. https://doi.org/10.1080/19325037.2019.1687365

**Reviewer: #1**

**Comments to the Author**

**I am interested in your topic because it is very interesting. I have found some issues regarding your manuscript that needs to be addressed.
1. In the methods part, you have used mixed method (Quant.+Qual.). You better to specify which type of mixed method you have used(Explanatory sequential, exploratory sequential or concurrent mixed method etc.)**

Thanks for this comment, we agree that we should have been clear about which type of mixed method we used. We have edited this statement in our methods.

Line 94-95: This is a mixed method (Quant.+Qual., convergent design), pre-post study of the virtual MiM^®^ program among people living with dementia and care partners.

**2. You have used purposive sampling method to select your participants, you have to specify which type of purposive sampling method you used.**

Thank-you for your comment. I’m assuming that you are referring to the selection of participants to take part in the study. It does appear to be purposive sampling because participants had to be living with dementia or be a care partner of someone living with dementia to be eligible for the study. But, given that the Alzheimer Society of Ontario drew on their local networks and promoted the study through social media, we believe that it aligns closer with a convenience sampling method. We have made a note of this in the manuscript to make it clearer for our readers.

Line 108-109: People living with dementia and their care partners were identified through the participating Alzheimer Societies (convenience sampling).

**3. Minor comments:-You better to cite the tables and figures in your text in accordance with plos guideline (Tab1, Tab2 etc, Fig1, Fig2 etc.**

Thank-you for this comment. Table 1 didn’t have an in-text citation so we have made that addition.

Line 308-309: A detailed breakdown of participants’ demographic information is shown in table 1.

**Reviewer: #2**

**Comments to the Author**

**This is an interesting interventional study. However some of the information needs clarification and those are included as comments in the attached document . Titles of Table 1 and 2 need to be revised.**

Thank-you for your recommended revisions. We have revised the titles of tables 1 and 2 to make them clearer and comprehensible. Unfortunately, we didn’t receive the remainder of your comments in the attached document. We reached out to PLOS One about this and they hadn’t received the comments so we were not able to respond further.

Line 311-312: Table 1. Participant characteristics by participant type (people living with dementia, care partners), expressed as mean (standard deviation) or percent.

Line 364-365: Table 2. Program satisfaction among people living with dementia (n=54) and care partners (n=68). Higher scores indicate greater satisfaction.

**Reviewer: #3**

**Comments to the Author**

**Overview:
It is an interesting study no doubt on an important aspect of supporting people living with dementia. However, its methodology is not sound enough to be worthy of publication in PLOS.
The study is entirely based on subjective reporting both quantitatively and qualitatively.**

We focus on outcomes prioritized by Canada’s National Dementia Strategy and its implementation under the Public Health Agency of Canada Dementia Community Investment, which funded this implementation research. While the outcomes were reported using questionnaires, these reflect the prioritized outcomes (quality of life/wellbeing and health behaviours), which are typically reported with questionnaires. While physical activity may be captured objectively (by accelerometers), quality of life and wellbeing are inherently subjective constructs. Furthermore, the study was completed while COVID-19 was still prevalent in Ontario, Canada and two province-wide lockdowns took place during the study period. As a result, all assessments were completed through Zoom, which complicates the administration of objective measures especially as participants are across diverse geographies, though we acknowledge this would not be impossible with sufficient funding.

Our study has different strengths. Our outcomes are aligned with national priorities for people living with dementia. In addition, our study sample is relatively large for interventions among people living with dementia and their care partners. As our intervention in the ‘real-world’, results are more generalizable than if the intervention and evaluation had been conducted in a highly-controlled academic setting.

**Furthermore, we are not sure how patients with dementia were able to rate them accurately.**

People living with dementia have a broad range of cognitive and communication abilities. In this evaluation, all participants had sufficient ability to follow instructions and communicate for the purpose of the intervention and evaluation.

The DEMQOL has been validated for use in people living with dementia. To ensure that results from the DEMQOL were reliable, we also asked care partners to complete the DEMQOL-Proxy for the person they cared for. Given that the change in scores were very similar across both questionnaires, we believe that it does point to the results being reliable and valid.

While people living with dementia were asked to complete the DEMQOL without their care partner in the room, the International Physical Activity Questionnaire and Cognitive Leisure Activity Scale were completed at the beginning of the assessment while their care partner was present. This ensured care partners could provide their input if the individual living with dementia was having trouble recalling their activities over the past week. This ultimately ensured that the responses given by people living with dementia were accurate. We didn’t feel that this was clear in the original manuscript so we have also outlined this in the revised manuscript.

Line 151-156: While people living with dementia independently completed the dementia quality of life questionnaire, their care partners remained in the room when they completed the International Physical Activity Questionnaire and the Cognitive Leisure Activity Scale. This ensured care partners could provide their input if the individual living with dementia was having trouble recalling their activities over the past week.

**We are not sure whether the study was powered adequately to reach a statistical significance.**

Thanks for your comment and concern. While we reached our recruitment target for the study which was outlined in our grant application, we acknowledge we may not have sufficient power to detect change in all outcomes, depending on the magnitude of change in that construct as well as the sensitivity of the assessment used. We have noted this limitation, which specifically may have impacted detection of change in the Warwick and Social Provisions Scale, which show a trend towards improvements but did not meet statistical thresholds.

**There was no attempt at randomization or a control group to compare with. The sample was a convenient one and there were many non-responders. They may not have responded as they did not benefit from the program.**

While we agree that the study design is not ideal for supporting causation, our goal was not to evaluate efficacy but to evaluate real-world feasibility and effectiveness. To enrol people to a randomized control trial of a program that is publicly available would be challenging and may reflect an ethical dilemma given that we would be restricting access to a program that showed benefits in its in-person format.

Other elements that you point out can be conversely viewed as a strength. Our sample reflects the people who enrol in the program/intervention in the ‘real-world’ and so is more generalizable to program participants than if we recruited narrowly to an academic study.

With respect to non-responders, the only questionnaire that had more than a few non-responders was the satisfaction questionnaire for people living with dementia, where 54/83 participants responded. These people living with dementia indicated that they didn’t remember taking part in the program so couldn’t accurately complete the satisfaction questionnaire.

**Only the qualitative findings may have some value. I am not sure whether this is adequate for a publication in PLOS.**

We will leave the decision to the editorial team at PLoS One. However, we do believe our study has strengths. There are very few studies that examine the feasibility and effectiveness of virtual exercise programs among people living with dementia, and no other studies that examine the benefits of a program with both exercise and cognitive stimulation. Our sample is relatively large for exercise interventions among people living with dementia and their care partners, and represents the population that typically enrols in the Minds in Motion program so results have strong generalizability.
