# Supplementary material for: Enhancing Minds in Motion® as a virtual program delivery model for people living with dementia and their care partners
Source: PLoS One. 2024 Jan 19;19(1):e0291166. doi: 10.1371/journal.pone.0291166 (PMC10798436; doi:10.1371/journal.pone.0291166)
Supplement: Attachment — Submitted filename: Reviewer comments.docx [file pone.0291166.s005.docx]

Reviewer comments: Enhancing Minds in Motion as a virtual program delivery model for people living with dementia and their Care Partners

Overview:

It is an interesting study no doubt on an important aspect of supporting people living with dementia. However, its methodology is not sound enough to be worthy of publication in PLOS.

Methodology:

The study is entirely based on subjective reporting both quantitatively and qualitatively. Furthermore, we are not sure how patients with dementia were able to rate them accurately. We are not sure whether the study was powered adequately to reach a statistical significance.

There was no attempt at randomization or a control group to compare with. The sample was a convenient one and there were many non responders. They may not have responded as they did not benefit from the program.

Findings: Only the qualitative findings may have some value. I am not sure whether this is adequate for a publication in PLOS.

Thank you
